# The Impact of Digital Technology on the Physical Health of Older Workers: Protocol for a Scoping Review

**DOI:** 10.2196/59900

**Published:** 2024-09-26

**Authors:** Jeroen J A Spijker, Hande Barlın, Diana Alecsandra Grad, Yang Gu, Aija Klavina, Nilufer Korkmaz Yaylagul, Gunilla Kulla, Eda Orhun, Anna Ševčíková, Brigid Unim, Cristina Maria Tofan

**Affiliations:** 1 Centre d'Estudis Demogràfics Bellaterra Spain; 2 Department of Economics Gebze Technical University Gebze Turkey; 3 Department of Public Health Faculty of Political, Administrative and Communication Sciences Babeș-Bolyai University Cluj-Napoca Romania; 4 Department of Work, Employment, Management & Organization University of Leicester Leicester United Kingdom; 5 Laboratory of Sports and Nutrition Research Riga Stradins University Riga Latvia; 6 Department of Health Promotion and Rehabilitation Lithuanian Sport University Kaunas Lithuania; 7 Gerontology Department, Faculty of Health Sciences Akdeniz University Antalya Turkey; 8 Department of Health and Caring Sciences Faculty of Health and Social Sciences Western Norway University of Applied Sciences Forde Norway; 9 College of Business American University Bulgaria Blagoevgrad Bulgaria; 10 Faculty of Social Studies Masaryk University Brno Czech Republic; 11 Department of Cardiovascular, Endocrine-Metabolic-diseases and Aging National Institute of Health Rome Italy; 12 Psychology and Educational Sciences Department Gheorghe Zane Institute for Economic and Social Research Romanian Academy Iasi Romania; 13 Department of Sociology, Social Work and Human Resources Faculty of Philosophy and Social-Political Sciences Alexandru Ioan Cuza University Iasi Romania

**Keywords:** digital tools, digital technology, digitalization, physical health, mobility, vision loss, musculoskeletal disorders, migraine, older workers, older population, aging, scoping review, mobile phone

## Abstract

**Background:**

Digital technologies have penetrated most workplaces. However, it is unclear how such digital technologies affect the physical health of older workers.

**Objective:**

This scoping review aims to examine and summarize the evidence from scientific literature concerning the impact of digital technology on the physical health of older workers.

**Methods:**

This scoping review will be conducted following recommendations outlined by Levac et al and will adhere to the PRISMA-ScR (Preferred Reporting Items for Systematic Reviews and Meta-Analysis extension for Scoping Reviews) guidelines for reporting. Peer-reviewed papers written in English will be searched in the following databases: MEDLINE, Cochrane, ProQuest, Web of Science, Scopus, APA PsycInfo, and ERIH PLUS. The web-based systematic review platform Covidence will be used to create a data extraction template. It will cover the following items: study and participant characteristics, health measures, digital tool characteristics and usage, and research findings. Following the Population, Concept, and Context (PCC) framework, our review will focus on studies involving older workers aged 50 years or older, any form of digital technology (including teleworking and the use of digital tools at work), and how digital technologies affect physical health (such as vision loss, musculoskeletal disorders, and migraines). Studies that focus only on mental health will be excluded. Study selection based on title and abstract screening (first stage), full-text review (second stage), and data extraction (third stage) will be performed by a group of researchers, whereby each paper will be reviewed by at least 2 people. Any conflict regarding the inclusion or exclusion of a study and the data extraction will be resolved by discussion between the researchers who evaluated the papers; a third researcher will be involved if consensus is not reached.

**Results:**

A preliminary search of MEDLINE, Epistemonikos, Cochrane, PROSPERO, and JBI Evidence Synthesis was conducted, and no current or ongoing systematic reviews or scoping reviews on the topic were identified. The results of the study are expected in April 2025.

**Conclusions:**

Our scoping review will seek to provide an overview of the available evidence and identify research gaps regarding the effect of digital technology and the use of digital tools in the work environment on the physical health of older workers.

**International Registered Report Identifier (IRRID):**

PRR1-10.2196/59900

## Introduction

Aging is a multifaceted process characterized by extensive intra- and interindividual differences, often conceptualized within theoretical frameworks as a dynamic balance between physiological advantages and limitations. Connected with this view, physical health has been described succinctly as “soundness of body” [1] and can be defined as a “dynamic state, the process of preserving and developing its biological, physiological and mental functions, optimal work capacity and social activity with the maximum life expectancy” [2], has been a major concern not only in the context of general aging but also aging in the workplace, especially with the extension of the working life and retirement age [3]. Physical health for older workers goes beyond maintaining the ability to perform job-related tasks effectively and includes prevention and management of age-related conditions, and ensuring overall well-being. Indeed, significant physiological changes have effects on the capabilities of older workers. These involve sensory function, muscle function, cardiovascular and respiratory function, neurological function, and immune response [4]. Nonetheless, despite the inevitable age-related physiological decline, a significant proportion of older workers demonstrate adaptation abilities, facilitating the maintenance of work performance [5].

As the global workforce ages and digital technologies increasingly penetrate the occupational landscape (through practices such as working from home, blended work, teleworking, and the use of digital apps), understanding the impact of digital technology on the physical health of older workers has emerged as a critical area of research. According to the latest European Union data from 2020, the share of older workers aged 55 years and older in the total number of employees increased from 12% to 20% between 2004 and 2019 [6]. At the same time, 21.6% of employees older than 55 years of age reported more than 2 work-related physical health problems [7].

With the global advancement of digital technology, especially in recent decades, the labor market and traditional work processes have undergone significant changes. New job roles and work conditions have emerged, introducing new physical and psychological requirements [8] that come with advantages and disadvantages. For instance, digital tools offer flexible arrangements such as working from home and diminish reliance on physically demanding tasks, thereby lowering the risk of musculoskeletal injuries, but only if workstations are ergonomically prepared, and regular breaks are taken [9]. However, the digitalization of work also introduces a spectrum of challenges that can adversely affect the physical health of older workers, including prolonged screen exposure leading to eye strain and headaches and extended periods of sitting without any physical activity, thereby increasing the risk of cardiovascular diseases and stress induced by the expectation of constant connectivity, which might lead to spinal, postural and other types of muscular disorders [9-11]. Furthermore, the inability to cope with technological innovation is a health risk that affects the work ability of older workers. Hence, providing continuous training or regular monitoring of biometric and physical health information [12,13], maintaining physical fitness, managing chronic diseases, and adapting workplaces are recommended to improve the safety and health of older workers and to support their productivity. Likewise, digital health coaching programs also assist older employees in maintaining health during the transition to retirement, potentially influencing physical health [14].

Against the aging of the workforce and the digital transition of the work environment, despite some emerging research and reviews in recent years focusing on understanding the psychological consequences of the digitalization of workplaces in terms of techno-stress [15,16], burnout, and mental strain [12,17,18], as well as on digital technologies for health and disease management [7,13,14,19-24], to the best of our knowledge, there is no existing systematic or scoping review that focuses on how older workers are affected physically by the digitalization of their tasks and workplaces. In this regard, this scoping review protocol aims to address the existing research gap in understanding the current scientific literature that examines various physical health consequences (positive or negative) of digitalizing work environments among older workers.

## Methods

### Guidance Frameworks

Based on the first methodological framework for conducting scoping reviews by Arksey and O’Malley, this scoping review will be conducted following the recommendations outlined by Levac et al [25] to guarantee a systematic and coherent process. Levac et al advocate proceeding by describing the following stages: (1) identifying the research question, (2) identifying relevant studies, (3) selecting studies, (4) charting and collating the data, and (5) summarizing and reporting the results. To report our findings, we will also adhere to the PRISMA-ScR (Preferred Reporting Items for Systematic Reviews and Meta-Analysis extension for Scoping Reviews) guidelines for reporting [26]. The PRISMA-ScR checklist for the scoping review will be reported in an appendix, and the PRISMA-ScR checklist for this protocol can be found in Multimedia Appendix 1.

### Protocol and Registration

This protocol was written before we performed the full electronic literature search (we searched for existing scoping reviews and pilot-tested different search terms; more details below). After the peer-review process the study was registered at the Open Science Framework [27].

### Stage I: Identifying the Research Question

We posed the research question: How do digital technologies in the workplace affect the physical health of older workers? As there are many ways in which digital technology can affect an older worker’s health (from vision loss to musculoskeletal disorders), there are many types (from working online from home to the use of robotics in a car assembly line); we also have the following subquestions: (1) What are the most common physical health issues that older workers have to manage as a result of digital technologies in the workplace? (2) What industries most affect older workers’ health as a result of the use of digital technology?

Based on our research questions, we defined the Population, Concepts, and Context (PCC) criteria of interest to clarify the focus of the scoping study and establish an effective search strategy (see also [25]; Textbox 1): To summarize the main points reflecting the PCC criteria: regarding the population, our review will focus on studies involving older workers aged 50 years or older.

The concept of interest is the implicit (eg, working from home) or explicit (eg, software systems, communication platforms, document management systems, lighting control systems, and automated and robotic systems) use of any type of digital technology within the work environment to execute job-related tasks. In terms of context, we are specifically interested in examining how digital technologies impact physical health issues such as vision loss, musculoskeletal disorders, migraines, and cardiovascular diseases. Studies focusing solely on mental health impacts will be excluded from this review.

**Definitions of the inclusion criteria regarding Population, Concepts, and Context in the scoping review.**

**Population: older workers**

- Older workers include study participants employed at the moment of the study.
- Study participants must be aged 50 years or older. When only age ranges are analyzed, the age of 50 should be included in the youngest age category (eg, 45-54). While ongoing debates exist regarding what age defines older workers [28], we have opted to include individuals 50 or older. This decision is based on the increasing presence of this cohort in the labor market, their likelihood of remaining in the workforce longer than previous generations, and the need to recognize the diversity within this demographic group. People in their 50s may have varying career trajectories, skill sets, and motivations for remaining in the workforce. By defining older workers as those aged 50 years and older, organizations can more effectively cater to the unique needs and experiences of this diverse and increasing group of older individuals in the labor market.
- If age is treated as a continuous variable rather than analyzed as categories, the sample also has to have participants aged 50 years or older; that is, younger ages are only permitted if older ages are also represented in the study.

**Concept: digital technologies**

- Digital technologies refer to data manipulation, storage, transmission, and processing in binary data [29]. It allows for the interaction with stored data using electronic devices (eg, computers and microprocessors). Digital data can be stored in various digital storage media (eg, hard drives, solid-state drives, memory cards, and cloud storage). Furthermore, digital technology also enables the transmission of data over digital communication networks (eg, the internet, local area networks, and wireless networks).
- Digital technologies are defined as any type of digital tool or device used in the context of (creative) production, that is, studies or study results that look at the effect of digital technologies not related to work (eg, for health management) are excluded.
- Digital technologies include the use of computers at home for work (eg, teleworking) as well as more recent digital technologies (eg, apps), but only if they are used for work purposes.
- Studies will be excluded if working from home does not involve digital tools (eg, only landlines are used).

**Context: physical health**

- Any physical health outcome is accepted.
- Mental health outcomes are excluded unless mentioned in combination with a physical health outcome.
- Studies will be excluded if the health outcome is not associated with an effect of the use of digital technologies in the work sphere.

### Stage II: Identifying Relevant Studies

We follow the JBI Manual for Evidence Synthesis [30] for our search strategy. For the protocol, the databases PubMed (Multimedia Appendix 2), PROSPERO, and JBI Evidence Synthesis were searched on April 15, 2024. Still, no current or underway systematic reviews or scoping reviews were identified (the 4 results shown are not related to the topic of our interest).

For the scoping review, we will search for peer-reviewed studies in the following databases: MEDLINE, Cochrane, ProQuest, Web of Science, Scopus, APA PsycINFO, and ERIH PLUS. The web-based systematic review platform Covidence will be used to import the references and remove duplicates. The search terms to be used are: (physical adj (health* OR condition* OR issue* OR impairment* OR fitness OR wellbeing OR (well adj being) OR integrit* OR state* OR stress) OR disease* OR vision OR mobility OR obes* OR overweight OR “Body Mass Index”) AND (digital OR app* OR web OR internet OR tech* OR (social adj media) OR chat OR online* OR cyber OR virtual OR computerized OR computerised OR electronic OR ICT) AND ((old* or elder* or ageing or ageing or senior*) adj1 (work* OR employee* OR profession* OR labor OR labour OR colleague* OR staff* OR cowork* OR personnel)).

In constructing our search strategy, we used the asterisk “*” symbol as an end-of-root-word-truncation mark and the Boolean operator “OR” to encompass a broad range of related physical health conditions, digital technologies, and terms referring to older workers. In addition, we used the adjacency operators “adj” and “adj1” to ensure that closely related terms appearing contiguously in the literature are captured, thereby enhancing the specificity of our search results and enabling a comprehensive inclusion of relevant studies.

A preliminary search conducted on April 18 and 19, 2024, for MEDLINE, Cochrane, and Epistemonikos provided 299, 260, and 5 references, respectively, before deduplication (more details in Multimedia Appendix 3).

In our final search, we will restrict our search to papers written in English and exclude any gray literature.

### Stage III: Study Selection and Eligibility Criteria

Once the references (excluding duplicates) are imported onto the Covidence platform, the first phase of the study selection will be the title and abstract screening. This is then followed by the full-text review to select the relevant studies for the main and secondary research questions of our scoping review. Finally, during the last phase, a data extraction template will be created in Covidence and used to facilitate the extraction of relevant information from the previously selected studies. This template will encompass items on participant demographics (age and gender), type of worker (occupation or employment branch), the specific digital technology examined and amount of usage, physical health outcome variables, and fundamental research findings derived from the selected studies.

Given the sheer number of papers likely to be retrieved during the first phase, most, if not all, co-authors will be involved in the selection process during each phase, whereby each paper will be revised by 2 people. Furthermore, when Covidence detects a conflict regarding the inclusion or exclusion of a study, this is then resolved by a third person.

In addition, regular online meetings will be held with the whole team to discuss any issues during the different study selection phases.

The eligibility for a study to be included will be based on the PCC and other criteria shown in Textbox 2. Note that there are no inclusion or exclusion criteria regarding the year of publication or the country of study. The former is because no previous scoping review on the precise topic has yet been published in a peer-reviewed journal, while any exclusion of territories would have to be justified.

**Eligibility criteria for the scoping review.**

**Inclusion criteria**

- Population: older workers (50+ included in study)
- Concept: digital technologies related to work
- Context: physical health outcomes
- Setting: nonclinical and in the work sphere
- Study type: original studies with any design or data type (quantitative and qualitative)
- Publication status: published in a peer-reviewed journal
- Publication language: English
- Full-text available

**Exclusion criteria**

- Population: younger workers (50+ not included in the study)
- Concept: digital technologies not related to work (eg, for health management)
- Context: nonphysical health outcomes (eg, mental health)
- Setting: clinical and not in the work sphere
- Study type: other study types (eg, protocols, narrative reviews, or systematic reviews)
- Publication status: published without peer review, dissertations, books, conference papers, letters, and editorials
- Publication language: written in a language other than English
- Full-text not available

### Stage IV: Charting the Data

The information from each selected publication, obtained from the final data extraction phase, will be summarized in a table. The main outcomes of interest are provided in Item 11 of the PRISMA-ScR checklist (see Multimedia Appendix 1).

### Stage V: Collating, Summarizing, and Reporting Results

In accordance with the recommendations outlined by Levac et al [25], the fifth stage of our methodology consists of 3 distinct steps: (1) analyzing research findings, encompassing both descriptive numerical summary analysis and qualitative thematic analysis; (2) evaluating the research findings to extract outcomes aligned with the research question, which are then reported narratively; and (3) interpreting and discussing the findings in relation to additional research questions, practical applications, and policy implications.

In addition to narrative reporting, tables will be used to provide a structured overview of the key findings. The PRISMA-ScR [26] guidelines will be adhered to ensure systematic reporting of the results.

In addition, we will assess the quality of studies using the Mixed Methods Appraisal Tool [31], whereby any discrepancies will be resolved through consensus between the reviewers.

## Results

We devised a comprehensive search strategy to identify papers on physical health issues associated with the use of digital technologies in the workplace. The outcomes of our inquiry will be disseminated through a scoping review. Consequently, the selection process for publication will be delineated using flowcharts, while the data extracted from our research will be organized in tables and expounded upon in a narrative summary. Subsequently, the summarized findings will endeavor to address the research question, “How do digital technologies in the workplace affect the physical health of older workers?” and the following subquestions: (1) what are the most common physical health issues that older workers have to manage as a result of using digital technologies at the workplace; and (2) what industries most affect older workers’ physical health as a result of applying digital technologies?

## Discussion

### Preliminary Findings

The scoping review outlined in this protocol will lay the groundwork for a comprehensive research initiative examining the impact of digital technologies on the health of older workers. Building upon the proposed work in the scoping review, there is potential to harness innovative technological solutions and evaluations to promote enhanced well-being and productivity among older workers in various work settings. Furthermore, we anticipate that the results of this scoping review will provide methodological insights and direction for exploring the integration of adaptive features for digital technology in the context of older workers. The findings from the scoping review will be shared through peer-reviewed scientific journals and conference presentations, contributing to the advancement of knowledge in this crucial area.

### Limitations

Our selection criteria restrict the inclusion of papers presenting empirical evidence published in English, potentially biasing the study pool toward research from Western countries as well as the physical health effects of digital technology used. Therefore, readers should exercise caution when interpreting the findings, considering the varying quality and applicability of the included studies.

The term “digital technology” was chosen as the subject heading for its relevance to our research scope. In developing our search strategy, we worked closely with an information specialist to pilot test a variety of terms, both subject headings and text words, aiming for comprehensive coverage. Subject headings are part of a controlled vocabulary that helps standardize the indexing of studies, while text words can vary greatly depending on the author’s choice of language. This variability in text word usage may affect the inclusiveness of the search results. For instance, authors may use different terminology to describe similar concepts, or a term might have different connotations in different regions. Despite a thorough approach and expert consultations, we must acknowledge the possibility of not capturing all pertinent studies due to the dynamic nature of terminology in this rapidly evolving field.

### Comparison With Previous Work

Our scoping review focuses on identifying digital technologies that impact the physical health of older workers and discerning which job types are most influenced by the integration of these technologies. The integration of digital technologies into the workplace offers potential benefits by automating physically demanding tasks and optimizing work processes, which could potentially reduce physical strain on older workers [32,33]. However, despite the frequent use of technologies like smartphones, laptops, and tablets among older individuals, comprehensive studies exploring the direct links between digital work environments and physical health outcomes among older workers are scarce.

Previous scoping and systematic reviews have predominantly examined the mental impacts of digital technology at work, such as those on mental health outcomes listed in Table 1. The focus was largely on psychotherapeutic interventions using digital tools, with an emerging interest in technologies such as extended reality. A comprehensive overview of the effectiveness of digital and technological interventions in mental health and well-being is provided by De Witte et al [34]. Similarly, Seberini et al [35] reviewed the impact of information and communication technologies (ICT) on older workers, particularly focusing on strategies to reduce the digital divide and technostress, and found how the rapid adoption of digital tools during the COVID-19 pandemic increased technostress among older workers as well as feelings of marginalization among older adults with a lack of technological skills, which also impacted their mental and physical health. Li [36], on the other hand, highlights how digital technologies were effectively used to address mental health issues among older workers during the COVID-19 pandemic, providing support through various web-based and mobile-based platforms. Overall, the reviews collectively suggest that while digital technologies hold significant potential for improving mental health outcomes in workplace settings, they also discussed the dual nature of digital technology in workplaces, where it can either exacerbate stress and mental health issues or be a tool for promoting well-being.

While there is extensive research on digital work’s mental health impacts, our review focuses on the less explored physical health implications for older workers. Substantial causal evidence regarding how digital technologies affect their physical health is notably scarce.

Therefore, the anticipated outcomes of our scoping review are expected to elucidate dimensions considered pertinent to health promotion and disease prevention, particularly in supporting and maintaining the physical health of the aging workforce. This review will underscore the significance of mitigating occupational health risks, emphasizing the crucial influence of various factors, including physical, ergonomic, and psychosocial elements, on work-related health outcomes.

**Table 1:** Selected systematic reviews on digital technologies for older people’s mental health.

Review citation	Population age (older people, years)	Digital technologies	Health outcomes
Li [36]	>50	eHealth and remote support	Reduction in depression, stress, and anxiety
De Witte et al [34]	50-55	Workplace digital interventions	Workability and health maintenance
Seberini et al [35]	≥60	Information and communication technologies	Technostress

### Conclusions

This scoping review will be the first to offer a comprehensive overview of physical health effects resulting from the use of digital technologies in the workplace. The findings of this scoping review will serve as foundational knowledge for understanding the impact of digitalization on the health and well-being of older workers, informing future research directions and potential interventions aimed at promoting the health of this demographic in evolving work environments.

## Appendix

Multimedia Appendix 1 PRISMA-ScR (Preferred Reporting Items for Systematic Reviews and Meta-Analyses extension for Scoping Reviews) checklist.

Multimedia Appendix 2 Search results of scoping reviews of the effect of digitalization on the physical health of older workers (no relevant hits).

Multimedia Appendix 3 Pilot test 1 of search terms of studies on the effect of digitalization on the physical health of older workers using Cochrane, Medline and Epistemonikos.

## Abbreviations

ICT: information and communication technologies
PCC: Population, Concept, and Context
PRISMA-ScR: Preferred Reporting Items for Systematic Reviews and Meta-Analyses extension for Scoping Reviews
