# Supplementary material for: The Impact of Digital Technology on the Physical Health of Older Workers: Protocol for a Scoping Review
Source: JMIR Res Protoc. 2024 Sep 26;13:e59900. doi: 10.2196/59900 (PMC11467605; doi:10.2196/59900)
Supplement: Multimedia Appendix 1 [file resprot_v13i1e59900_app1.pdf]

Spijker J, Barlın H, Grad DA, Gu Y, Klavina A, Korkmaz Yaylagul N, Kulla G, Orhun E, Sevcikova A, Unim B, Tofan CM (25 July 2024). The impact of digital technology on the physical health of Older Workers: Protocol for a Scoping Review. JMIR Research Protocol 2024(13):e59900. DOI: 10.2196/59900.

Multimedia Appendix 1: PRISMA-ScR checklist.

Table of contents

Study details according to the PRISMA-ScR checklist 2
Item 1. Title 2
Item 2. Abstract 2
Item 3. Rationale 2
Item 4. Objectives 2
Item 5. Protocol and registration 3
Item 6. Eligibility criteria 3
Item 7. Information sources 3
Item 8. Search 3
Item 9. Selection of sources of evidence 3
Item 10. Data charting process 3
Item 11. Data items 4
Item 12. Critical appraisal of individual sources of evidence 5
Item 13. Summary measures 5
Item 14. Synthesis of results 5
Item 15. Risk of bias across studies 5
Item 16. Additional analyses 5
Items 17-26: Results / Discussion 5
Item 27: Funding 5
References 5

Study details according to the PRISMA-ScR checklist

This study employs a scoping review design, adhering to the Preferred Reporting Items for Systematic Reviews and Meta-Analyses Extension for Scoping Reviews (PRISMA-ScR) checklist [1]. The current document is a short summary of the protocol that is currently undergoing peer-review in the journal JMIR Research Protocol.

Item 1. Title

The impact of digital technology on the physical health of Older Workers: Protocol for a Scoping Review.

Item 2. Abstract

Background: Digital technologies have penetrated most workplaces. However, it is unclear how such digital technologies affect the physical health of older workers.

Objective: This scoping review aims to examine and summarize the evidence from scientific literature concerning the impact of digital technology on the physical health of older workers.

Methods: This scoping review will be conducted following recommendations outlined by Levac et al. and will adhere to the PRISMA-ScR (Preferred Reporting Items for Systematic Reviews and Meta-Analysis Extension for Scoping Reviews) guidelines for reporting. Peer-reviewed articles written in English will be searched in the following databases: MEDLINE, Cochrane, Proquest, Web of Science, Scopus, APA PsycInfo and ERIH PLUS. The web-based systematic review platform Covidence will be used to create a data extraction template. It will cover the following items: study and participant characteristics, health measures, digital tool characteristics and usage and research findings. Following the Population, Concept, and Context (PCC) framework, our review will focus on studies involving older workers aged 50 years or above, any form of digital technology (including teleworking and the use of digital tools at work) and how digital technologies affect physical health (such as vision loss, musculoskeletal disorders, and migraines). Studies that focus only on mental health will be excluded. Study selection based on title and abstract screening (first stage), full-text review (second stage) and data extraction (third stage) will be performed by a group of researchers, whereby each article will be reviewed by at least two people. Any conflict regarding the inclusion or exclusion of a study and the data extraction will be resolved by discussion between the researchers who evaluated the papers; a third researcher will be involved if consensus is not reached.

Results: A preliminary search of MEDLINE, Epistemonikos, Cochrane, Prospero, and JBI Evidence Synthesis was conducted and no current or underway systematic reviews or scoping reviews on the topic were identified. The results of the study are expected in April 2025.

Conclusions: Our scoping review will seek to provide an overview of the available evidence and identification of research gaps regarding the effect of digital technology and the use of digital tools in the work environment on the physical health of older workers.

Item 3. Rationale

The aim of this scoping review is to locate and synthesize evidence on the impact of digital technology on the physical health of older workers.

Item 4. Objectives

The objective of this scoping review is then to identify the scientific literature that addresses the impact of digital technology on the physical health of older workers. Hence, the scoping review will focus on the following topics:

1) Study design and focus.

2) Digital technology type

3) Employment setting

4) Physical health effects

5) Evidence gaps in this field.

Item 5. Protocol and registration

After the peer-review process the study was registered at the Open Science Framework (https://osf.io/dj34a)

Item 6. Eligibility criteria

The eligibility criteria for this scoping review are based on the Population, Concept and Context (PCC) criteria:

Inclusion criteria

1. Population: older workers (50+ included in study)
2. Concept: digital technologies related to work
3. Context: physical health outcomes
4. Setting: nonclinical and in the work sphere
5. Study type: original studies with any design or data type (quantitative and qualitative)
6. Publication status: published in a peer-reviewed journal
7. Publication language: English
8. Full-text available

Exclusion criteria:

1. Population: younger workers (50+ not included in study)
2. Concept: digital technologies not related to work (e.g. for health management)
3. Context: non-physical health outcomes (e.g. mental health)
4. Setting: clinical and not in the work sphere
5. Study type: other study types (e.g. protocols, narrative reviews or systematic reviews)
6. Publication status: published without peer-review, dissertations, books, conference papers, letters, editorials.
7. Publication language: written in a language other than English
8. Full-text not available

Item 7. Information sources

The information sources for this scoping review include the following international bibliographic databases, which are used to identify the most relevant systematic and scoping reviews as well as scientific studies: MEDLINE, Cochrane, Proquest, Web of Science, Scopus, APA PsycInfo, ERIH PLUS, Prospero, and JBI Evidence Synthesis.

Item 8. Search

The syntax for the electronic search was developed and calibrated throughout March-April 2024 with the help from an experienced Research Librarian. The full search strategy will be reported in the scoping review.

Item 9. Selection of sources of evidence

After importing references and removing duplicates on COVIDENCE, a web-based systematic review platform, the study selection begins with title and abstract screening, followed by a full-text review to identify articles relevant to the main and secondary research questions of our scoping review. A pre-developed data extraction template will then be used to collect data from the selected articles, covering participant demographics, examined digital technologies, physical health outcomes, key findings, and policy implications. Due to the expected high volume of articles, a team of researchers will conduct the selection process, with each article reviewed by two individuals. Conflicts over article inclusion are resolved by a third reviewer. Regular online team meetings will ensure smooth progression through the different phases of article selection.

Item 10. Data charting process

To organize and synthesize data effectively to extract information pertinent to our research question the data extraction will be conducted entirely within COVIDENCE as it offers robust features for collaboration and

comprehensive data handling. The data-charting form for data coding used for this will be developed by JS and calibrated within the team. For each extracted article the data will be coded independently by 2 researchers and final consensus will be reached during discussion.

Item 11. Data items

Information from the following list of data items and their description will be retrieved from the articles to address the objectives of this scoping review. The name of the reviewer will be written at the top (for internal use only):

**Item**	**Description**
Author (APA style)	If 1 author: Author Surname (year). If 2 authors: Author and Author (year). If 3+ authors: First author et al. (year)
Year of data collection	
N (number of participants)	
N of participants invited	How many people were interviewed or had questionnaires sent to them in total, no matter if they responded?
N in follow-ups	For example, in intervention or randomized control studies: How many participants responded in a follow-up (e.g., after 1 year)?
Country	
Type of study	Quantitative, qualitative, mixed
Sampling method	How were participants recruited for the nresearch?
Data collection method	What type of method was used? e.g. semi-structured interview, in-depth interview, face-to-face questionnaire/survey, telephone survey, online survey, mail survey
Data recording method	Pencil-paper, online, audio, video, , secondary data, other
Type of population	e.g. employees, employers, general population
Type of workers	Occupation/Employment branch
Age categories used in analysis/results	If no age categories were analyzed, the age range of the sample
Study includes both young and older workers?	Studies with young people may only be included if older workers are also represented and analysed (at least 50 years old)
Includes both older adults and older workers?	Studies with older adults who do not work may only be included if older workers are also analysed
Gender/sex used in analysis/results	If participants are described for each gender, check each box (”female”, ”male”). If participants are described as whole check ”total f+m”
Digital tool	Name / describe the digital tool that was used
Digital tools characteristics	Implicit (e.g., remote work, hybrid), Explicit (e.g., apps, digital tools used at work), both (implicit and explicit)
Type of physical health	What physical health outcome was studied?
Instruments	Describe instruments/measures/assessments used for the variables digital tools and physical health. How were the variables assessed?
Main findings	Describe relevant findings for our research goals/questions
General effects	Positive, negative, mixed, no effects

Item 12. Critical appraisal of individual sources of evidence

Although a critical appraisal of individual studies is usually not conducted in a scoping review [1], we will assess the quality of studies using the Mixed Method Appraisal Tool (MMAT) [2] whereby any discrepancies will be resolved through consensus between the reviewers.

Item 13. Summary measures

Not applicable for scoping reviews.

Item 14. Synthesis of results

The charted data will be placed in a table and narratively synthesized.

Item 15. Risk of bias across studies

Not applicable for scoping reviews.

Item 16. Additional analyses

Not applicable for scoping reviews.

Items 17-26: Results / Discussion

Not applicable at the protocol stage.

Item 27: Funding

The research was partially financed through the COST Action CA21107 “Work inequalities in later life redefined by digitalization” (DIGI-net) that is supported by the European Cooperation in Science and Technology (COST) (https://www.cost.eu/actions/CA21107/).

References

1. Tricco AC, Lillie E, Zarin W, O'Brien KK, Colquhoun H, Levac D, et al. PRISMA Extension for Scoping Reviews (PRISMA-ScR): Checklist and Explanation. Annals of Internal Medicine. 2018;169(7):467-73. https://doi.org/10.7326/M18-0850.
2. Hong QN, Fàbregues S, Bartlett G, Boardman F, Cargo M, Dagenais P, et al. The Mixed Methods Appraisal Tool (MMAT) version 2018 for information professionals and researchers. Education for information. 2018;34(4):285-91.
