# Supplementary material for: The Impact of Digital Technology on the Physical Health of Older Workers: Protocol for a Scoping Review
Source: JMIR Res Protoc. 2024 Sep 26;13:e59900. doi: 10.2196/59900 (PMC11467605; doi:10.2196/59900)
Supplement: Multimedia Appendix 2 [file resprot_v13i1e59900_app2.pdf]

Gmail

Protocol Scoping Review Digi health

1 message

Gu, Yang <.................>
To: jeroen spijker <jspijker@ced.uab.es>

15 April 2024 at 23:38

Dear Jeroen,

My university has a PubMed account, so I did a preliminary search. The search results(4 results) are as shown in the attached picture. I hope it is helpful.

1 Cite Share

A systematic scoping review of community-based interventions for the prevention of mental ill-**health** and the promotion of mental **health** in **older** adults in the UK.

Lee C, Kuhn I, McGrath M, Remes O, Cowan A, Duncan F, Baskin C, Oliver EJ, Osborn DPJ, Dykxhoorn J, Kaner E, Walters K, Kirkbride J, Gnani S, Lafortune L; NIHR SPHR Public Mental Health Programme.

Health Soc Care Community. 2022 Jan;30(1):27-57. doi: 10.1111/hsc.13413. Epub 2021 May 14.

PMID: 33988281 Review.

BACKGROUND: Mental **health** concerns in **older** adults are common, with increasing age-related risks to **physical health**, mobility and social isolation. Community-based approaches are a key focus of public **health** strategy in the UK, and may reduce th ...

2 Cite Share

Patient Portals to Support Palliative and End-of-Life Care: Scoping Review.

Ingle MP, Valdovinos C, Ford KL, Zhou S, Bull S, Gornail S, Zhang X, Moore S, Portz J.

J Med Internet Res. 2021 Sep 16;23(9):e28797. doi: 10.2196/28797.

PMID: 34528888 Free PMC article. Review.

BACKGROUND: Although patient portals are widely used for **health** promotion, little is known about the use of palliative care and end-of-life (PCEOL) portal tools available for patients and caregivers. ...As medical care increasingly moves toward virtual platforms, future re ...

3 Cite Share

Digital Health Coaching Programs Among **Older** Employees in Transition to Retirement: Systematic Literature Review.

Stara V, Santini S, Kropf J, D'Amen B.

J Med Internet Res. 2020 Sep 24;22(9):e17809. doi: 10.2196/17809.

PMID: 32969827 Free PMC article. Review.

OBJECTIVE: This systematic literature review aimed to explore studies on **digital health** coaching programs for **older workers** that followed a user-centered design approach and evaluated their effectiveness in providing **older** adults with guidance f ...

4 Cite Share

Effectiveness of community-based interventions for **older** adults living alone: a systematic review and meta-analysis.

Kim I, An H, Yun S, Park HY.

Epidemiol Health. 2024;46:e2024013. doi: 10.4178/epih.e2024013. Epub 2024 Jan 3.

PMID: 38228090 Free article.

Of the 2,729 identified studies, 9 met the criteria for inclusion in this review. Independent variables

Best wishes,

Yang
