# Supplementary material for: The Impact of Digital Technology on the Physical Health of Older Workers: Protocol for a Scoping Review
Source: JMIR Res Protoc. 2024 Sep 26;13:e59900. doi: 10.2196/59900 (PMC11467605; doi:10.2196/59900)
Supplement: Multimedia Appendix 3 [file resprot_v13i1e59900_app3.pdf]

Database: Ovid MEDLINE(R) ALL <1946 to April 18, 2024>
Search Strategy:

1 (physical adj (health* or condition* or issue* or impairment* or fitness or wellbeing or (well adj being) or integrit* or state*)).ti,ab. (63475)
2 Disease/ (69309)
3 disease*.ti,ab. (4840202)
4 (vision* or mobility or obes* or overweight or (body adj mass adj index)).ti,ab. (904353)
5 exp Overweight/ (281984)
6 1 or 2 or 3 or 4 or 5 (5664473)
7 (digital or app* or web or internet or tech* or (social adj media) or chat or online* or cyber or virtual or computerized or computerised or electronic or ICT).ti,ab. (10512561)
8 ((old* or elder* or ageing or ageing or senior*) adj1 (work* or employee* or profession* or labor or labour or colleague* or staff* or cowork* or personnel)).ti,ab. (5531)
9 6 and 7 and 8 (299)

Search Name:
Date Run: **19/04/2024 04:57:46** Cochrane
Comment:

ID Search Hits
#1 (physical NEXT (health* or condition* or issue* or impairment* or fitness or wellbeing or (well next being) or integrit* or state*)):ti,ab,kw (Word variations have been searched) 15416
#2 MeSH descriptor: [Disease] explode all trees 9831
#3 (disease*):ti,ab,kw (Word variations have been searched) 552182
#4 (vision* or mobility or obes* or overweight or (body NEXT mass NEXT index)):ti,ab,kw (Word variations have been searched) 151006
#5 MeSH descriptor: [Overweight] explode all trees 24883
#6 #1 or #2 or #3 or #4 or #5 670846
#7 (digital or app* or web or internet or tech* or (social NEXT media) or chat or online* or cyber or virtual or computerized or computerised or electronic or ICT):ti,ab,kw (Word variations have been searched) 680382
#8 ((old* or elder* or ageing or ageing or senior*) NEAR/1 (work* or employee* or profession* or labor or labour or colleague* or staff* or cowrk* or personnel)):ti,ab,kw (Word variations have been searched) 1100
#9 #6 and #7 and #8 260

Epistemonikos

Search history

#	Query	Date	Remove
4	(title:("physical health" OR disease OR vision* OR mobility OR obesity OR overweight OR "body mass index") OR abstract:("physical health" OR disease OR vision* OR mobility OR obesity OR overweight OR "body mass index")) AND (title:(digital OR app OR web OR internet OR "social media" OR chat OR online* OR cyber OR virtual OR computerized OR computerised OR electronic OR ICT) OR abstract:(digital OR app OR web OR internet OR "social media" OR chat OR online* OR cyber OR virtual OR computerized OR computerised OR electronic OR ICT)) AND (title:("older workers" OR "older employees" OR "older colleagues" OR "older staff") OR abstract:("older workers" OR "older employees" OR "older colleagues" OR "older staff"))	19-04-2024 12:03:32 +02:00	✖
3	"older workers" or "older employees" or "older colleagues" or "older staff"	19-04-2024 12:03:09 +02:00	✖
2	digital or app or web or internet or "social media" or chat or online* or cyber or virtual or computerized or computerised or electronic or ICT	19-04-2024 12:02:13 +02:00	✖
1	"physical health" or disease or vision* or mobility or obesity or overweight or "body mass index"	19-04-2024 12:01:30 +02:00	✖

5 hits
